# Bend and Moisture Effects on the Performance of a U-Shaped Slotted Wearable Antenna for Off-Body Communications in an Industrial Scientific Medical (ISM) 2.4 GHz band

**DOI:** 10.3390/s19081804

**Published:** 2019-04-15

**Authors:** Rocio Sanchez-Montero, Pablo-Luis Lopez-Espi, Cristina Alen-Cordero, Juan-Antonio Martinez-Rojas

**Affiliations:** Department of Signal Theory and Communications, Escuela Politécnica Superior, Universidad de Alcalá, Campus Universitario, Ctra. de Madrid a Barcelona km 33.600, 28805 Alcalá de Henares, Spain; pablo.lopez@uah.es (P.-L.L.-E.); cristina.alen@uah.es (C.A.-C.); juanan.martinez@uah.es (J.-A.M.-R.)

**Keywords:** wearable antennas, WBAN, ISM, moisture, bend

## Abstract

In recent years, the study and design of wearable antennas have been empowered given the success of Wireless Body Area Networks (WBAN) for healthcare and medical purposes. This work analyses a flexible textile antenna whose performance can be optimised by the careful selection of the substrate thickness of the textile material, and by varying the antenna’s geometrical shape. After considering these parameters, several arrangements of antennas were simulated using the Computer Simulation Technology software (CST). The results of the simulations were compared to the experimental prototypes manufactured on a flexible felt material for a range of thicknesses and curvatures of the antenna substrate. Such antenna designs can be utilised in off-body communications and ISM applications.

## 1. Introduction

In the last decade, a top priority for most governments has been to reduce healthcare expenditure due to larger numbers of elderly people. In fact, the US Bureau of Census has described that in [1] 2025, elderly people will have doubled from 35 to 70 million only in the USA. In the EU, almost one fifth the population was aged over 65 in 2017, and this figure is forecast to be about 29% by 2050 according to Eurostats [2].

**Figure 1:**
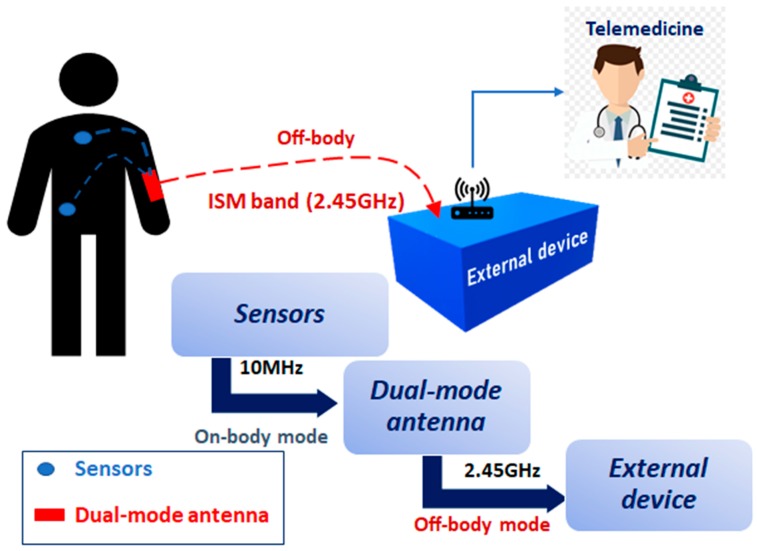
Proposed health monitoring system [16]. ISM band: Industrial Scientific Medical band.

The tendency of most research works has focused on developing new user-friendly devices made with smart textile materials. The development of antennas with textile materials heralds a new era for using non-invasive sensors in clothing to allow real-time health monitoring [3,4]. The definition of Wireless Body Area Networks (WBAN), given by the IEEE 802.15.6 group in 2010 [5], marked an important advance in health care for medical proposes because it supports a wide range of medical and consumer electronics (CE) applications. Thus, WBAN allow patients to be monitored in real-time with no restrictions to ordinary life [6,7], as well as other activities like sports. Standard IEEE 802.15.6 [8] defines several frequency bands for WBAN applications. Of them all, the Industrial Scientific Medical Band (ISM: 2.4 GHz and 5.8 GHz) stands out. The requirements for WBAN sensors should be little power use, a low profile, good compactness, easy to integrate into fabrics and should avoid any body influence on the antenna’s properties as much as possible. Based on the physical properties of the textile materials described by recent research works [9], the development of suitable wearable electronics and antennas to be integrated into fabrics has been achieved [10,11,12,13]. These integrable wireless sensors may be located in, on or around the human body. According to the distribution of these wearable sensors, it is possible to meet a requirement in personal healthcare systems: human body communication to collect medical data (on-body) and human-to-human body communication to exchange data with outside networks (off-body) [14,15]. The proposed health monitoring system described in [16] is shown in Figure 1.

One of the most suitable antennas used in humans for off-body communications is the U-shaped antenna. The first definition of such an antenna was provided by Huynh and Lee [17] in 1995 as a single-layer wideband patch antenna. In [18], it is firmly established that a U-shaped antenna can provide impedance bandwidths in excess of 20%. This U-slot rectangular patch antenna etched on a finite grounded substrate fulfils thin profile and small size requirements. Furthermore, this structure does not present any grating-lobe problems when used to form part of an array [19,20]. These characteristics allow these antennas to be attaching inside clothes, and can even touch human skin to transmit biophysical signals from a body to the external device.

It is important to guarantee proper antenna performance when it is placed in different body locations, such as the legs, arms or wrists. The contact established between the antenna and skin can modify its frequency range values [21,22]. The most relevant drawbacks are due to the bending of fabrics or to substrate wetting. Bending effects have been widely studied by varying antenna dimensions for a fixed textile substrate thickness [23,24,25,26,27]; although increased substrate thickness can lead to a less comfortable prototype, it is a simple cheap way to improve antenna robustness. On the contrary, it is very difficult to find studies that have dealt with the effect of dampness on antenna performance. Rizwan et al. in [11] merely mentioned the possibility of running a humidity test in a future work. Jalil et al. [27] studied the effect on the performance of an antenna subjected to bending and wetting for GSM, WIFI and WLAN was analysed for a fixed low thickness Denim substrate. They state that it is important to choose a suitable substrate with low absorption to ensure that the antenna can perform well under wet conditions after carrying out some test for different water absorption values. Their study was focused on establishing a maximum absorption limit and concludes on the possibility of running a humidity test in a future work. In [28], the return loss increasing in a single feed rectangular-ring textile antenna for ISM band due to bending effect is studied for two curvature values. In [29], five selected textile materials, applied as antenna substrate, were analysed in a different humidity condition. In this paper, Hetleer et al. reveals the influence of the moisture in the resonance frequency. The study varies the water content for a fixed substrate thickness. Apart from that, Scarpello et al. in [30] have carried out a detailed study related to an antenna array. They analysed the influence of the relative humidity and the bending effects, when the array is placed on-body and covered by different textile layers taken into account the stability of the return losses and mutual coupling characteristics. The present work analyses how the antenna performance can be modified by varying substrate thickness for different bend curvatures and by carefully modifying the moisture of a given textile.

The paper is arranged as follows: antenna design, characteristics and variables are defined in Section 2. Section 3 provides details of the simulation results and the experiments carried out to measure the antenna. Finally, Section 4 draws the final conclusions about the research performed in this paper.

## 2. Materials and Methods

**Figure 2:**
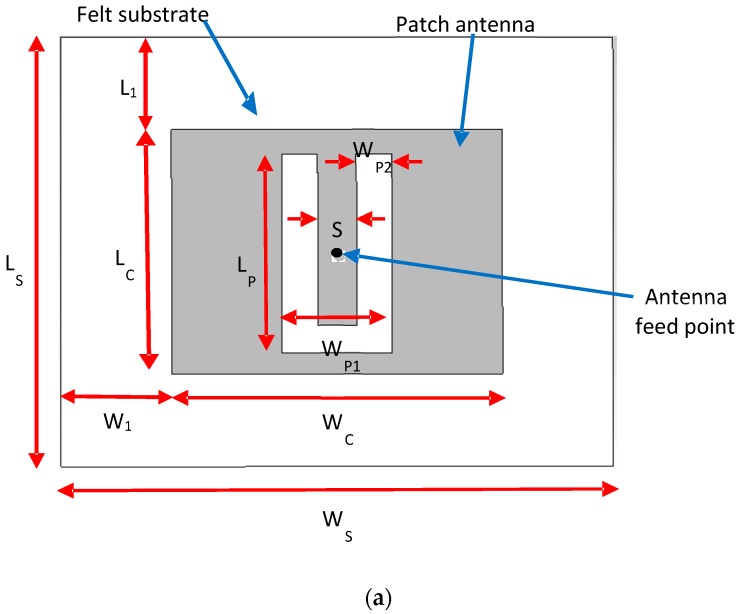

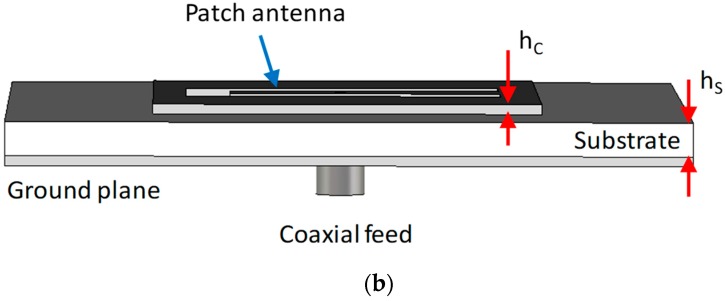
Geometry of the flexible textile antenna with a U-shaped aperture. (**a**) Top view, (**b**) side view.

The flexible antenna considered herein comprises a centre-fed patch with a U-shaped aperture near the feed point, as illustrated in Figure 2. The dimensions of the proposed design are provided in Table 1 below. The U aperture and the feeding point are placed symmetrically inside the patch antenna. The first prototype of this patch antenna, defined by Huynh and Lee [17], was given as a linearly polarised antenna with an impedance bandwidth exceeding 30% for an air substrate of about 0.08λo. New studies have subsequently shown that U-slot patch antennas are capable of providing other functions, including wideband characteristics, dual-band and triple-band operations [31,32,33] and circular polarisation with a wide axial ratio bandwidth [34,35]. For the equivalent substrate thickness, they usually offer a wider bandwidth than a conventional rectangular or circular patch [18]. Only this feature makes them suitable to be incorporated into a flexible textile antenna design for ISM applications.

The first step to start the design phase consists in simulating the U-shaped patch antenna with a textile material. For this purpose, a flexible felt (ε_r_ = 1.3, acting as the dielectric) and a flexible copper tape (acting as the conductor) were used. The dielectric and conductor thicknesses were set at 6 and 0.035 mm, respectively.

**Figure 3:**
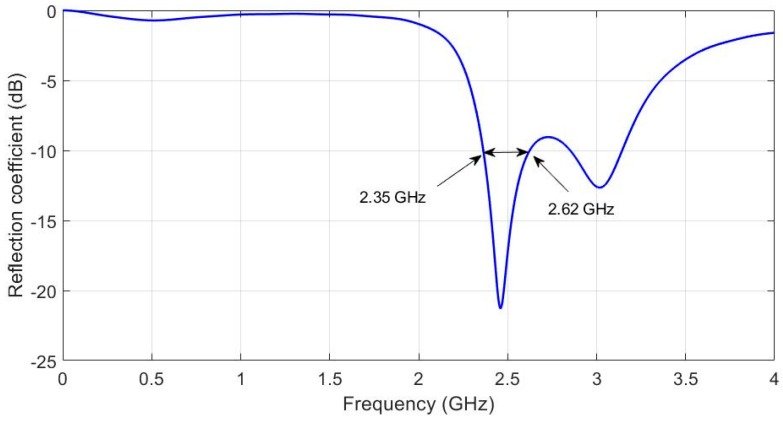
Reflection coefficient simulated response of the original antenna U-shaped patch prototype to a textile substrate.

The software used in the design phase to simulate antenna performance was CST Microwave Studio™ [36]. Figure 3 shows the reflection coefficient (S_11_) obtained with the original antenna values indicated in Table 1.

The reflection coefficient illustrated in Figure 3 shows that the main resonant frequency is 2.45 GHz and the bandwidth is about 2.35 to 2.62 GHz (11,02%). In accordance with the specification of Standard IEEE 802.15.6 described in [8], antenna performance met the necessary requirements to be used for ISM communications in an off-body health monitoring system. However, it is crucial to take into account that the main functionality of wearable systems is their implementation into garments. Thus, they will suffer bending and wetting, which can modify the reflection coefficient response. In order to check the antenna’s robustness and its capability to meet ISM requirements, an exhaustive analysis was carried out, as shown in the next section.

## 3. Results and Discussion

The antenna design and parametric tolerance analysis were performed using the time domain solver in CST Microwave Studio™. Simulations were carried out from 0 Hz to 4 GHz using 15 lines per wavelength and an accuracy of −50 dB.

### 3.1. Substrate Thickness Variation. Simulated Results

**Figure 4:**
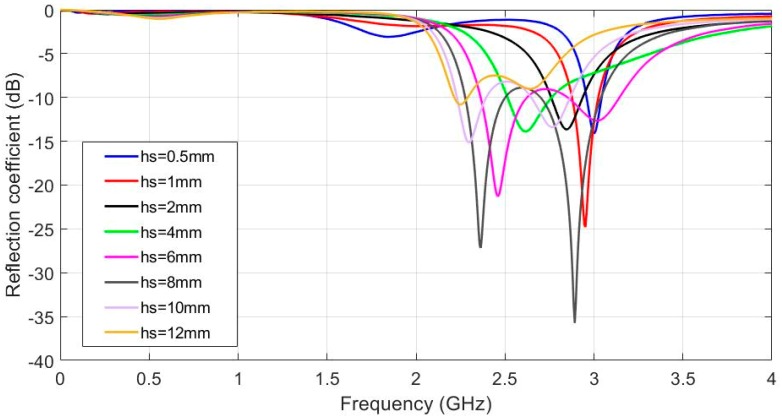
Simulated reflection coefficient for the different substrate thickness values, h_S_ from 0.5 mm to 12 mm, h_C_ = 0.1 mm, ε_r_ = 1.3 and tanδ = 0.058.

Initially, the influence of substrate thickness on the antenna reflection coefficient was analysed for a dry flat antenna. Figure 4 shows the results for substrate thicknesses from hs = 0.5 mm to hs = 12 mm. Other parameters remained constant, according to Table 1. Conductor thicknesses h_C_ = 0.1 mm, ε_r_ = 1.3 and tanδ = 0.058 were considered. Based on this result, it was asserted that the greater the increase in substrate thickness, the lower the resonant frequency value. Resonant frequency varies from 3 GHz to 2.2 GHz as substrate thickness varies from 0.5 to 12 mm. For any hs values above 5 mm, a second resonance appeared at around 500 MHz beyond the main one. An intermediate thickness value of hs = 6 mm was selected for the ISM band.

### 3.2. Antenna Bending Variation. Simulated Results

**Figure 5:**
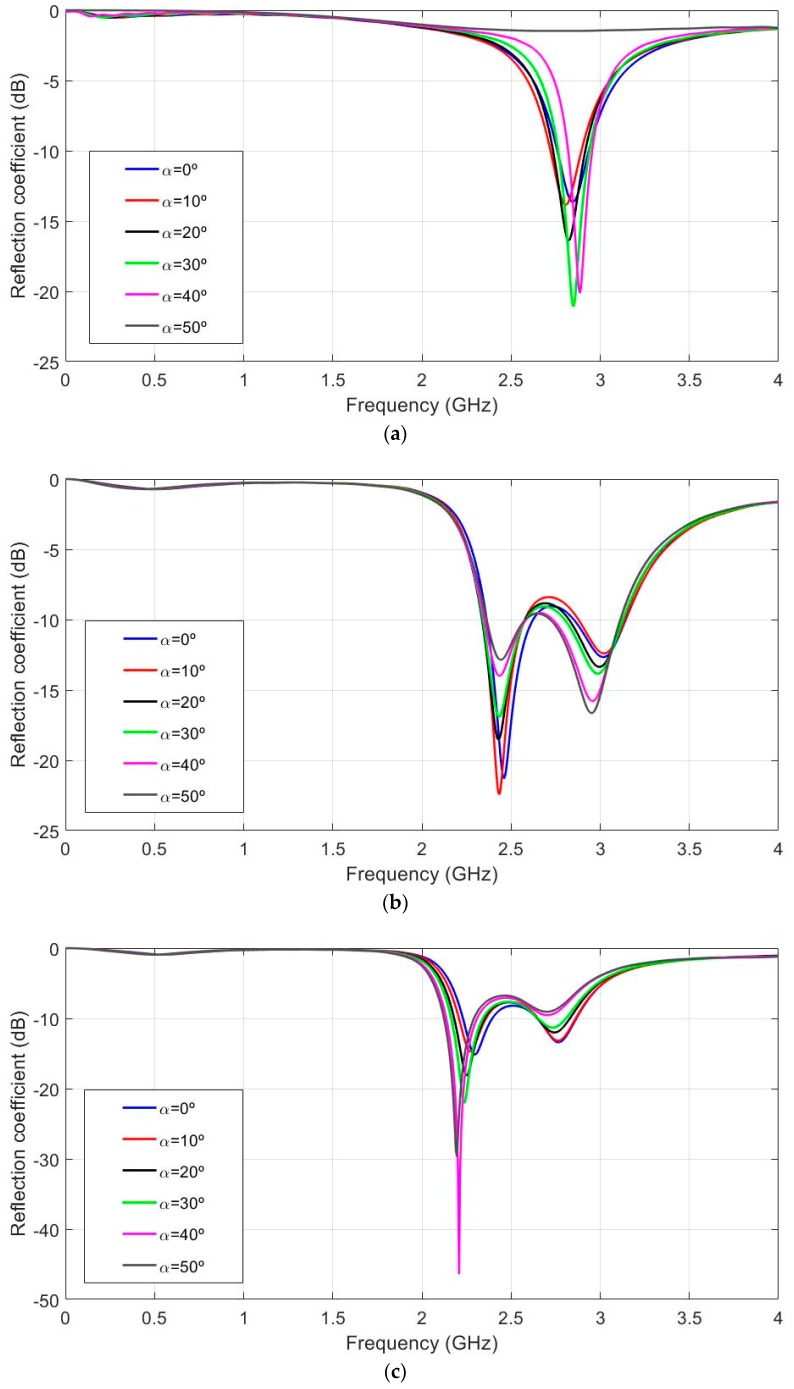
Simulated reflection coefficient for the different values of the curvature angles, α from 0° to 50° in 10° steps, εr = 1.3 and tanδ = 0.058. (**a**) hs = 2 mm, (**b**) hs = 6 mm and (**c**) hs = 10 mm.

According to the possible antenna location on the body (for example arm or wrist), the curvature effects in the H-Plane were simulated for different bending angles (α) from 0 to 50 degrees for the different substrate thickness values These bending angles are equivalent to 24.38, 30.48, 40.6, 69.8 and 121.9 mm of curvature radio values. These values were chosen as being representative to cover the arm curvature in a regular body. For the sake of simplicity, only the plots for the thicknesses of 2, 6 and 10 mm are presented in Figure 5a–c, respectively.

Figure 5a (hs = 2 mm) shows that the resonant frequency varied from 2.886 to 2.802 GHz (84 MHz shift) for the different bending values. The relative bandwidth of the flat antenna was 6.34%. Figure 5b (hs = 6 mm) shows that the resonant frequency varied from 2.430 to 2.460 GHz (30 MHz shift) for the different bending values. The relative bandwidth of the flat antenna was 11.02%. Figure 5c (hs = 10 mm) depicts how the resonant frequency varied from 2.196 to 2.298 GHz (102 MHz shift) for the different bending values. The relative bandwidth of the flat antenna was 6.96%. From these results, it can be stated that the intermediate substrate thickness values were less sensitive in frequency shift terms than higher or lower ones. For flat antenna performance, the best results in relative bandwidth terms were obtained for the intermediate thickness (hs = 6 mm). As substrate thickness increased, a noticeable resonance in the response was observed. The second resonant frequency displayed the same behaviour in shift frequency and relative bandwidth terms. This could lead to better relative bandwidth performance. Hence, further research may be conducted to increase the relative bandwidth by combining both resonances; however, this is beyond the scope of this research.

**Figure 6:**
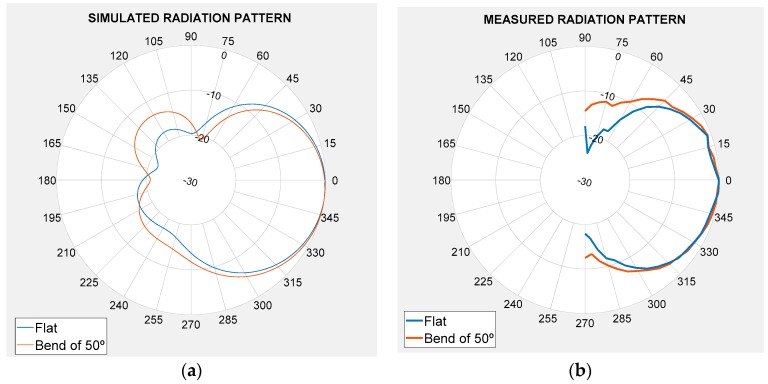
Radiation pattern of the 6 mm thickness antenna for flat and bend positions (α = 50°). (**a**) Simulation results and (**b**) measurement results.

To totally verify antenna performance, the radiation pattern was analysed. Figure 6 shows the plane H radiation pattern for the 6 mm dielectric thickness (hs = 6 mm). The radiation pattern was represented for hs = 6 mm because, in line with the results in Figure 5, this is the best case according to the relative bandwidth. Although the measured results will be discussed in a following section, for a better comparison, Figure 6 represents the simulated (a) and measured (b) radiation pattern for the flat case (blue) and for the worst bend (α = 50°) case (red) at 2.45 GHz.

According to the results in Figure 6, the antenna curvature effects did not modify the main radiation pattern characteristics. Apart from the radiation pattern study, another study about the radiation parameters was done in detail (see Table 2, Table 3 and Table 4).

The antenna bend effect led to a more marked gain decrease when the antenna thickness value was low; e.g., hs = 2 mm or hs = 4 mm. Another main consequence was deduced from the results in Table 2, Table 3, Table 4, Table 5 and Table 6; efficiency was not significantly modified. As regards the beam width data, the values were almost the same when the antenna was in the flat or the bend position at different angles. In Table 6 it is worth noting that when antenna thickness was low (hs = 2 mm) and the angle curvature was high, beam width increased up to 14°.

### 3.3. Moisture Influence. Simulated Results

**Figure 7:**
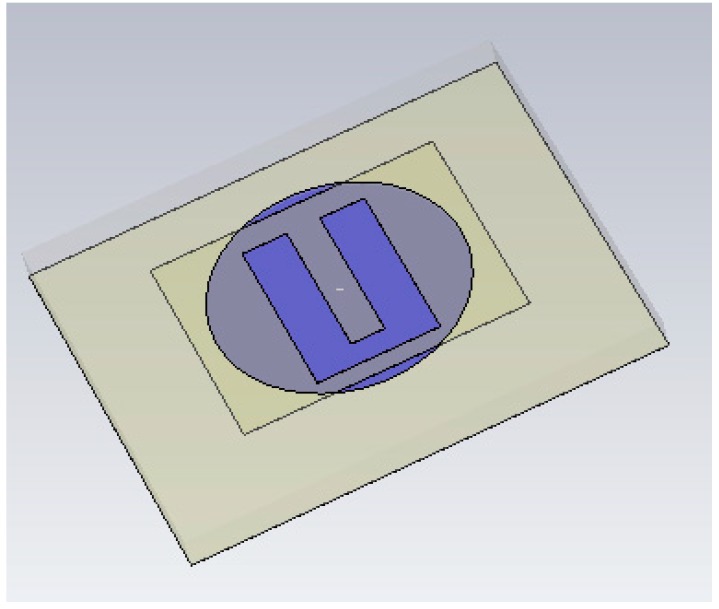
Prototype model in the CST Microwave Studio to analyse the moisture effect.

Most models of water migration (wicking) in porous and fibrous materials consider that the material is isotropic at the relevant scales and that the wicking process achieve a steady state when enough time has passed, due to the balance of forces between water weight and capillarity [37,38,39]. In our case, the fabric has paper-like properties and celulose fibres are oriented in random directions, thus the isotropic approximation is valid and a simple drop model is applicable. The size of the drop is smaller than the antenna surface and, due to the weight of the drop, water is not able to damp all the material, but only a volume just below the point of impact. This simple drop model is described in [37,38,39]. In [37,38], Starov et al., Bormashenko et al. and Masoodi et al. described models of wicking in paper-like porous media and calculated the height of liquid with time. A linear front was generally assumed so that only the height of the liquid column was determined after some time. In our case, the time after wetting was long enough to assume a steady state. Thus, in our model, the length of the water column increases as the water is being added to the fabric. The damped surface used for the simulation of the effect of moisture is represented in Figure 7. We have simulated several volume shapes: cylindrical, prismatic and finally, a conical volume of damped material was considered. The cone radius and height were considered a variable which was adjusted to fit the experimental added water volume as well as possible. Very big differences have been found between the different simulated shapes for the same water volume. The conical shape was the best when resonance frequency shifts and high order resonances were considered.

Table 5 shows the water content for the different experiments. The experimental water values used for measurements were taken into account in the simulations.

**Figure 8:**
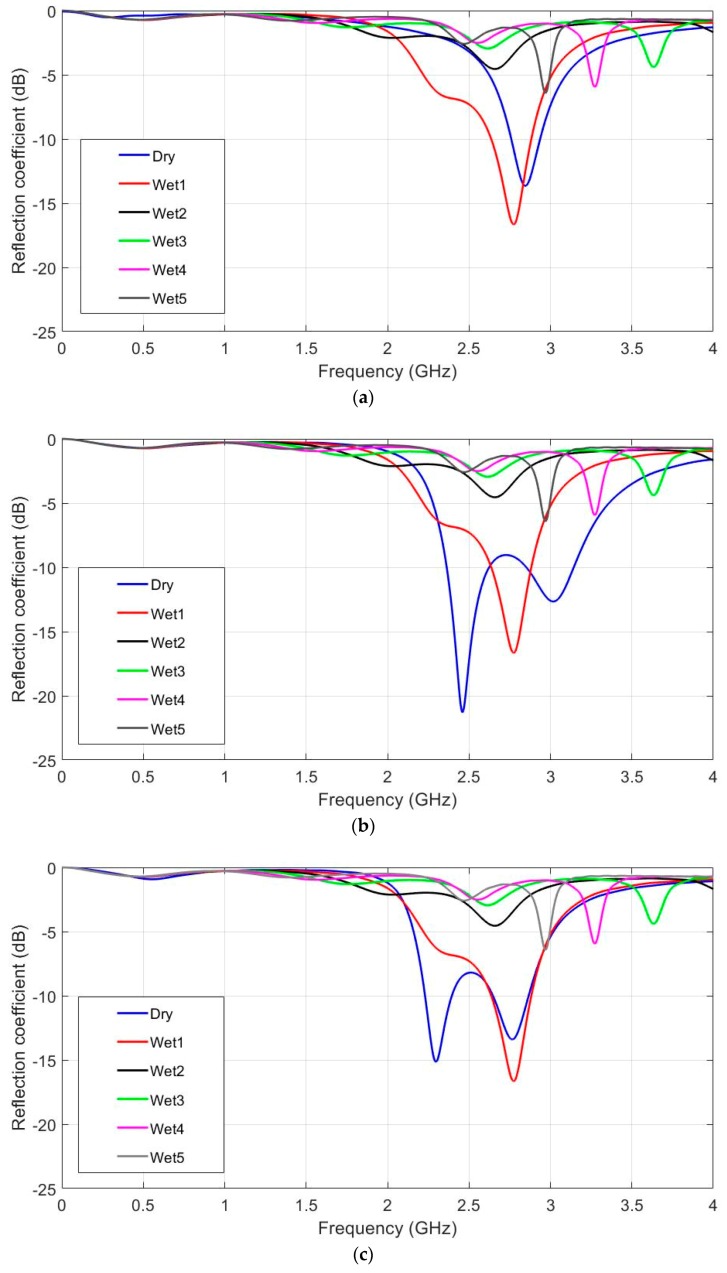
Simulated reflection coefficient for the different water concentration values. ε_r_ = 1.3 and tanδ = 0.058. (**a**) hs = 2 mm, (**b**) hs = 6 mm and (**c**) hs = 10 mm.

The moisture influence on antenna performance is shown in Figure 8, and hs = 2, 6 and 10 mm cases are represented in Figure 8a–c. The dry antenna was compared to five wet configurations. In Figure 8a (hs = 2 mm), the resonant frequency varies from 2.84 to 2.77 GHz for the dry and wet1 cases, and vanishes for the other cases. For example, the wet2 case is at 2.65 GHz, but the reflection coefficient value is higher than −10 dB. The second resonance appears for cases 2 to 5. This is due to the increase in the effective dielectric constant of water and felt as water content grows. Figure 8b (hs = 6 mm) shows that the resonant frequency varies from 2.46 to 2.76 GHz for the dry and wet1 cases. In fact, as the water content increases, the original resonance varies rapidly and high order resonances appear in the frequency range. For the other configurations, the results are similar to the hs = 2 mm case. It can be seen that water content, even for the wet1 case, makes antenna performance worse. Once again however, antenna optimisation could lead to a weaker moisture influence. In Figure 8c (hs = 10 mm), the resonant frequency varies from 2.30 to 2.77 GHz for the dry and wet1 cases. As in the previous case (hs = 6 mm), the second resonance dominates, but is more insensitive to the water influence. The water influence was negligible in the comparison of both the second resonances. For the other cases, the results were once again similar to the hs = 2 mm case. By way of conclusion, the proposed antenna better tolerated a small moisture content as antenna substrate thickness increased.

**Figure 9:**
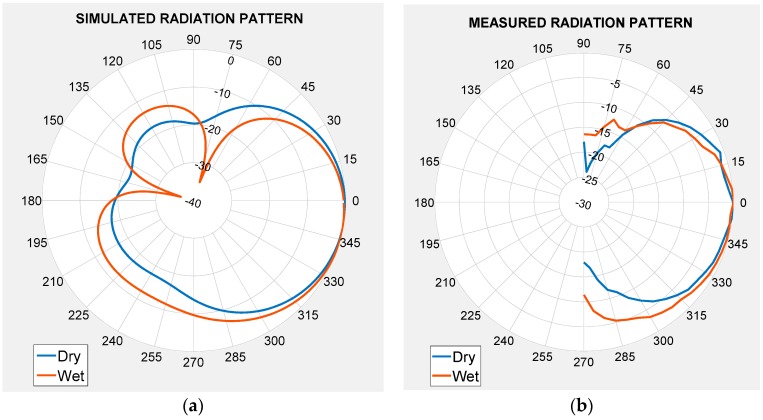
Radiation pattern of the antenna with hs = 6 mm for the dry and wet conditions (1.59 g of water). (**a**) Simulation results and (**b**) measurement results.

Besides the reflection coefficient, the radiation pattern of the antenna under the dry and wet conditions was determined. Figure 9 shows the comparison between the antenna radiation pattern for the dry condition and at the maximum water concentration (wet5 = 1.59 g of water). The pattern has been represented in both cases, dry (blue) and wet (red) conditions, at the resonant frequency: 2.45 GHz for the dry case and 3 GHz for the wet case, which was the most suitable according to the reflection coefficient represented in Figure 8b. In this figure, antenna thickness was fixed at 6 mm. For a better comparison, Figure 9b shows the measured radiation pattern.

Figure 9 depicts that the radiation properties in the main direction are the same no matter whether the antenna is dry or wet. The principal moisture influence on this wearable antenna is in the null. The minimum value of the radiation pattern is lower when the antenna is wet.

However, it is important to take into account the possible influence of the different amounts of water in the U-shaped antenna for different dielectric thicknesses. For this reason, a thorough analysis of the main radiation pattern characteristics was done, and the results are presented in Table 6, Table 7 and Table 8.

According to these results, the higher the water concentration, the lesser the antenna gain, and. the more antenna thickness is, the greater the gain decrease. However, the beam width values were not modified that much, only when hs = 10 mm and there was more water (1.21 g and 1.59 g). In these cases, beam width increased by 4°–5°, as shown in Table 8.

### 3.4. Measurement Results

**Figure 10:**
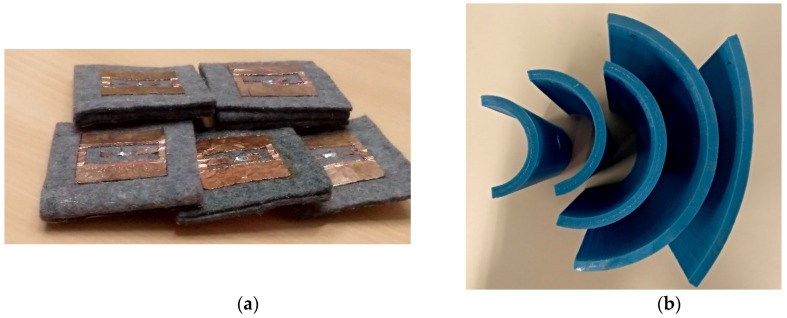

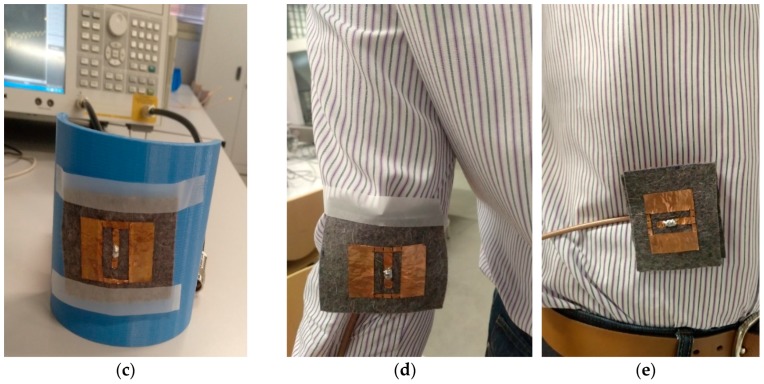
Measurement setup used to test the bend effect on textile antennas. (**a**) Antenna prototypes with different felt thicknesses, (**b**) three-dimensional (3D) bending structures, (**c**) Network Analyzer and (**d**,**e**) Arm and waist measurements.

**Figure 11:**
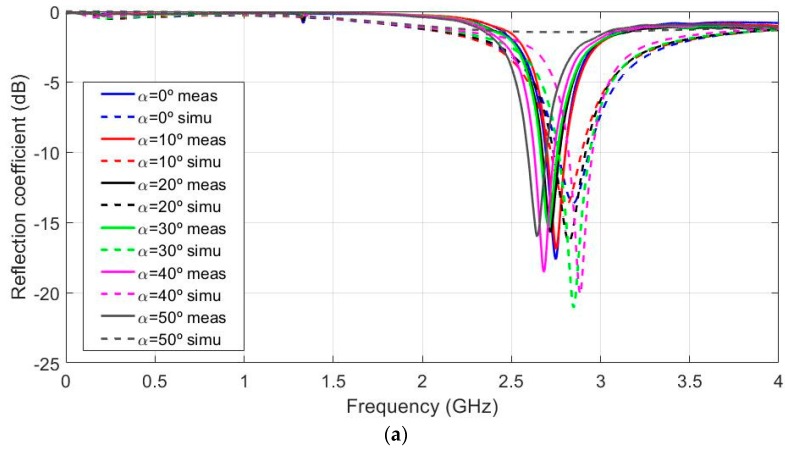

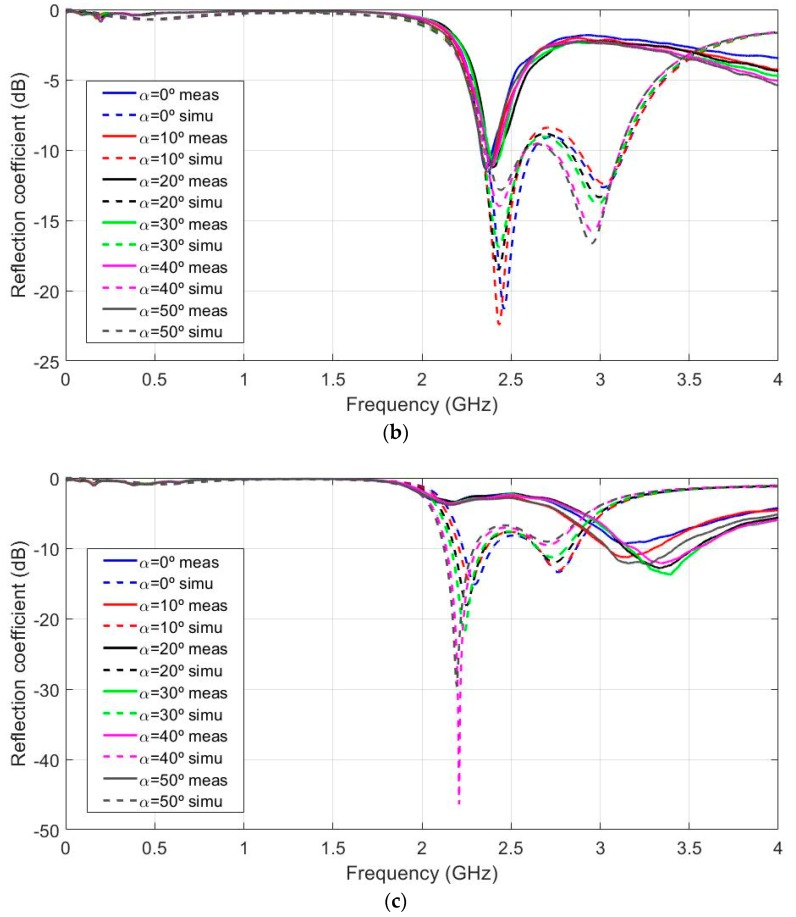
Comparison of the reflection coefficient measured and simulated with different values for the curvature angles, α from 0° to 50° in 10° steps. εr = 1.3 and tanδ = 0.058. (**a**) hs = 2 mm, (**b**) hs = 6 mm and (**c**) hs = 10 mm.

Figure 10a shows the different manufactured prototypes from hs = 2 mm to hs = 10 mm in steps of 2 mm. Antennas were stacked in different felt layers. For a better consistency, layers were glued using a solvent free glue stick. This technique was chosen to ensure the same dielectric properties for all the manufactured antennas, but it has probably also led to a kind of layered substrate instead of a strictly homogeneous one. A set of bending fixtures is shown in Figure 10b. They were built with a BQ Wibox2 three-dimensional (3D) printer [40]. Prototypes were measured using an E5071C Agilent Network Analyzer in a free space environment. Figure 10c represents the reflection coefficient measurement. The simulated and measured results for the different bending values are shown in Figure 11.

Once again for the sake of simplicity, only three representative results are shown (hs = 2, 6 and 10 mm) in Figure 11a–c, respectively. Measurements were plotted on a bold line and the previously discussed simulations were plotted using dash lines. The same colour was used for both measurements and simulations, and for the same bending angle. Measurements and simulations gave a proper fit, especially for thinner antennas (i.e., single or double layer prototypes); however, as the number of layer increases, the measured results suffer larger deviations from the simulated ones. Other difference between measurement and simulations was that the second resonance shown in the simulations was not noted in the measurements. This could be due to the differences between the stacked and the single layer felt dielectric. A better stacking model for simulation could require further research. On the contrary, measured and simulation results agree when the resonant frequency shift due to bending effects is considered. Measurements showed that thinner and thicker antennas underwent greater shifts than intermediate ones. This proves that optimising dielectric thickness is a key factor to achieve more robust designs.

**Figure 12:**
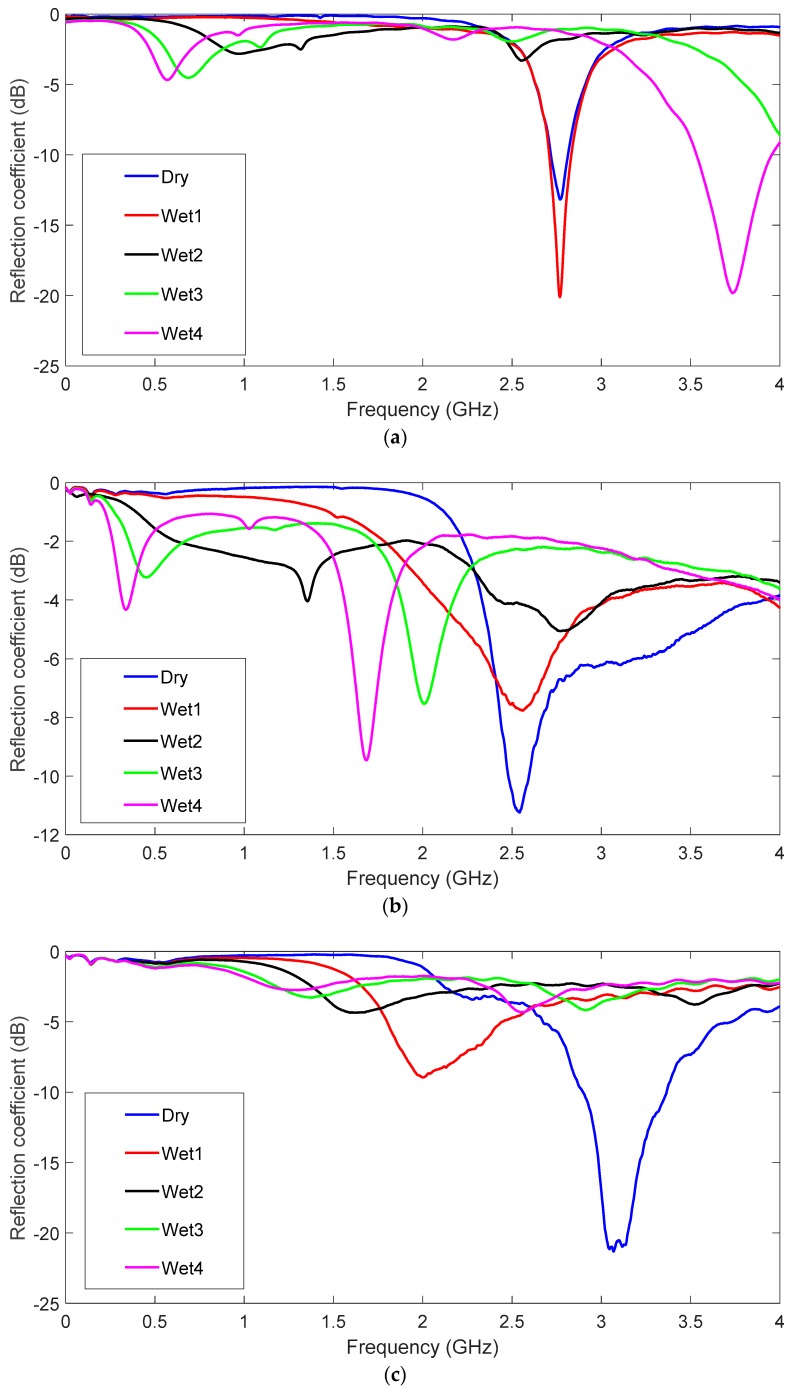
Measured Reflection coefficient for the different values of the water in the prototype with several felt thicknesses. (**a**) hs = 2 mm, (**b**) hs = 6 mm and (**c**) hs = 10 mm.

Moisture effects were also measured for the different prototypes. Water drops were homogeneously distributed all over the U-shape for the measurements. Antennas were weighed on a proper weighing scale to add the same water content to the different prototypes. Thinner and thicker antennas were strongly affected by the moisture influence, and resonant frequency rapidly turned to lower values, as seen in Figure 12a–c. According to the measurements, wet4 and wet5 cases are very similar, thus, for better readability, only five cases have been depicted: dry and wet1 to wet4 cases. The water vertical distribution in the felt was more homogeneous in thinner antennas. For the thinner substrates, small water values are enough to soak all the felt. On the contrary, as the substrate thickness increases, the supplied water amount is not enough to penetrate all the felt. This leads to a better agreement between measurements and simulations for the thinner prototypes. Conversely, the measurements and simulations made with thicker antennas did not provide such a good match in terms of the second resonance noted in the simulations. As shown in Figure 12b, the intermediate thickness values underwent lower frequency variations. This conclusion once again indicates introducing antenna thickness into the set of variables for further optimisation purposes.

**Figure 13:**
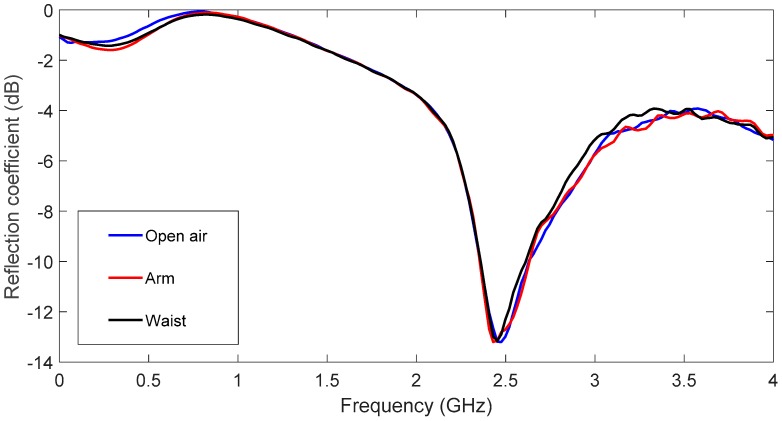
Reflection coefficient measured in the air and on the body.

Finally, in order to verify the body influence when the antenna is placed on it, two additional measurements have been carried out. Figure 10d shows the setup employed to take measurements. In the arm case, the antenna has been fixed using adhesive tape to get a proper bend. In the waist case, the antenna has been measured in a flat position. The open air and on-body (arm and waist) measurements are plotted in blue, red and black, respectively, in Figure 13. As can be seen, the body influence is negligible. This emphasizes the results shown in Figure 11b.

## 4. Conclusions

Herein, a detailed study of the performance of a U-Shaped aperture antenna with a textile substrate (felt) was conducted after considering the bending and moisture influences for different substrate thickness values. A set of antennas was simulated for the thickness substrate values from 0.5 to 12 mm, and five prototypes were manufactured for the thickness substrate values from 2 to 10 mm. The results show good agreement between simulations and measurements, especially for thinner antennas. The stacked layer manufacturing technique produced minor differences between measurements and simulations as substrate thickness increased. Thus, a more complex simulation layer model needs to be further investigated. Thinner antennas were more sensitive to the bend and moisture influences. Thicker antennas underwent wider variations when humidity changed; however, this could be due to the manufacturing technique, and also to the inhomogeneous water distribution into felt. Some models were tested, but their accuracy according to the experimental measurements was poorer as substrate thickness grew because water concentrated close to the felt surface. Further research on water distribution may be conducted too. The experimental results confirmed the strong influence of substrate thickness on antenna robustness. A disadvantage was increased substrate thickness because it could lead to a less comfortable prototype. Some humidity drawbacks could be overcome with an encapsulated design, but isolation would increase the design cost and would also lead to a less adaptable design.

## Figures and Tables

**Table 1:** Antenna design parameters.

Parameter	Value (mm)	Parameter	Value (mm)
L_S_	70	W_P1_	17
W_S_	85	W_P2_	5.5
L_C_	39.9	h_S_	6
W_C_	51	h_C_	0.035
S	6	L_1_	15.05
L_P_	32.5	W_1_	17

**Table 2:** Simulated antenna radiation pattern **gain (dBi)** for flat and bend positions at 2.45 GHz.

Dielectric Thickness (mm)	Flat	Bend α = 10°	Bend α = 20°	Bend α = 30°	Bend α = 40°	Bend α = 50°
hs = 2	8.176	8.040	7.595	6.615	3.179	3.307
hs = 4	8.271	8.261	8.169	8.044	7.788	7.353
hs = 6	8.326	8.340	8.290	8.241	8.163	8.067
hs = 8	8.339	8.355	8.314	8.282	8.236	8.177
hs = 10	8.278	8.286	8.241	8.214	8.164	8.114

**Table 3:** Simulated antenna radiation pattern **efficiency** for flat and bend positions at 2.45 GHz.

Dielectric Thickness (mm)	Flat	Bend α = 10°	Bend α = 20°	Bend α = 30°	Bend α = 40°	Bend α = 50°
hs = 2	0.1233	0.1089	0.08703	0.06889	0.06158	0.1427
hs = 4	0.3242	0.3244	0.3061	0.2840	0.2553	0.2241
hs = 6	0.4888	0.4947	0.4892	0.4777	0.4616	0.4493
hs = 8	0.6021	0.6106	0.6122	0.6093	0.6052	0.6015
hs = 10	0.6613	0.6679	0.6711	0.6733	0.6730	0.6731

**Table 4:** Simulated antenna radiation pattern **beam width (°)** for flat and bend positions at 2.45 GHz.

Dielectric Thickness (mm)	Flat	Bend α = 10°	Bend α = 20°	Bend α = 30°	Bend α = 40°	Bend α = 50°
hs = 2	72.5	72	71.6	71.7	121.9	86.4
hs = 4	72.2	72	71.9	71.7	71.4	71.2
hs = 6	71.9	71.9	71.9	71.9	71.8	71.8
hs = 8	71.7	71.8	71.9	72	72.1	72.2
hs = 10	71.8	71.9	72.1	72.3	72.6	72.8

**Table 5:** Water content used to simulate the moisture effects on different antenna samples.

Water Weight. Wet1 (gr)	Water Weight. Wet2 (gr)	Water Weight. Wet3 (gr)	Water Weight. Wet4 (gr)	Water Weight. Wet4 (gr)
0.24	0.53	0.86	1.21	1.59

**Table 6:** **Gain (dBi)** of the simulated radiation pattern of antenna performance for the dry and wet conditions at 2.45 GHz.

Dielectric Thickness (mm)	Dry	Wet (0.24 g of Water)	Wet (0.53 g of Water)	Wet (0.86 g of Water)	Wet (1.21 g of Water)	Wet (1.59 g of Water)
hs = 2	8.176	7.581	6.780	6.407	6.588	7.355
hs = 4	8.271	7.803	6.748	6.384	6.684	7.527
hs = 6	8.326	7.906	6.847	6.507	6.726	7.370
hs = 8	8.339	7.906	6.794	6.324	6.300	6.532
hs = 10	8.278	7.678	6.260	5.599	5.247	5.049

**Table 7:** **Efficiency** of the simulated radiation pattern of antenna performance for the dry and wet conditions at 2.45 GHz.

Dielectric Thickness (mm)	Dry	Wet (0.24 g of Water)	Wet (0.53 g of Water)	Wet (0.86 g of Water)	Wet (1.21 g of Water)	Wet (1.59 g of Water)
hs = 2	0.1233	0.1423	0.1876	0.2392	0.2661	0.2795
hs = 4	0.3242	0.3558	0.4501	0.5108	0.5328	0.5051
hs = 6	0.4888	0.6382	0.6186	0.6624	0.6692	0.6390
hs = 8	0.6021	0.6382	0.7215	0.7396	0.7462	0.7527
hs = 10	0.6613	0.7073	0.7762	0.7894	0.7955	0.7818

**Table 8:** **Beam width (°)** of the simulated radiation pattern of antenna performance for the dry and wet conditions at 2.45 GHz.

Dielectric Thickness (mm)	Dry	Wet (0.24 g of Water)	Wet (0.53 g of Water)	Wet (0.86 g of Water)	Wet (1.21 g of Water)	Wet (1.59 g of Water)
hs = 2	72.5	72.9	73.5	73.6	73.1	72.5
hs = 4	72.2	72.3	72.8	72.8	72.2	71.7
hs = 6	71.9	71.7	72.1	72	71.8	72
hs = 8	71.7	71.7	71.9	72.2	72.7	74.5
hs = 10	71.8	71.9	72.9	73.9	75.7	79.6
